# Surgical Site Infections in Patients of Periampullary Carcinoma Undergoing Delayed Bile Duct Division (COMBILAST) in Whipple’s Procedure: A Prospective Cohort Study

**DOI:** 10.3390/pathogens12030448

**Published:** 2023-03-13

**Authors:** Prakash Kumar Sasmal, Kallol Kumar Das Poddar, Tushar Subhadarshan Mishra, Pankaj Kumar

**Affiliations:** Department of Surgery, AIIMS, Bhubaneswar 751019, Odisha, India

**Keywords:** pancreaticoduodenectomy, Whipple’s procedure, surgical site infections, biliary contamination, aberrant right hepatic artery, accessory right hepatic artery, postoperative morbidity

## Abstract

Surgical site infections (SSIs) following a pancreaticoduodenectomy have been a significant cause of morbidity and even mortality. A modified sequence of the Whipple procedure, using the COMBILAST technique, may reduce SSIs and the patient’s hospital stay. This prospective cohort study included 42 patients undergoing Whipple’s pancreaticoduodenectomy for a periampullary malignancy. The modified sequence pancreaticoduodenectomy technique, COMBILAST, was used to estimate the incidence of SSI and explore other advantages. Of the 42 patients, seven (16.7%) developed superficial SSIs, and two patients (4.8%) had an additional deep SSI. Positive intraoperative bile culture had the strongest association with SSI (OR: 20.25, 95% CI: 2.12, 193.91). The mean operative duration was 391.28 ± 67.86 min, and the mean blood loss was 705 ± 172 mL. A total of fourteen (33.3%) patients had a Clavien–Dindo grade of III or higher. Three (7.1%) patients died of septicemia. The average length of a hospital stay was 13.00 ± 5.92 days. A modified sequence of the Whipple procedure, using the COMBILAST technique, seems promising in reducing SSIs and the patient’s hospital stay. As the approach is only a modification of the operative sequence, it does not compromise the oncological safety of the patient. Moreover, it has an added surgical advantage in reducing the chance of injury to the aberrant or accessory right hepatic artery.

## 1. Introduction

One of the most frequent maladies troubling the postoperative patient is surgical site infections (SSIs). It accounts for approximately 20% of all hospital-acquired infections (HAI) [1,2] as per the data from the National Surgical Quality Improvement Programme (NSQIP), which poses an additional burden on the healthcare system due to an increase in the consumption of antibiotics and wound care products and a longer length of hospitalization (LOH) [3]. The literature suggests that the incidence of SSI is even higher in patients undergoing pancreaticoduodenectomy, ranging from 20 to 50% [4,5,6]. 

The risk factors for developing SSI after hepatobiliary (HPB) surgery have been studied [7]. Bile leakage and pancreatic fistula formation are common causes of organ space infection after pancreatectomy [8]. The incidence of an organ space SSI occurring concurrently with a pancreatic fistula is 76% [7]. It is assumed to be because of a superadded bacterial infection in the background tissue; the damage is caused by autodigestion, and the mechanism is through the pancreatic enzymes. Preoperative biliary drainage was associated with increased intraoperative positive bile cultures and subsequent surgical site infective complications [9]. Preoperative biliary drainage promotes bile lithogenicity and favors colonization of the biliary tree with endogenous microorganisms. During bile-duct transection intraoperatively, the colonized bile can contaminate the peritoneal cavity and surgical wound, thus effectively converting a Class II (clean-contaminated) surgical environment into a Class III (contaminated) field. Owing to these factors, it is better to avoid routine preoperative biliary drainage [10]. 

The risk factors for a superficial SSI are different due to the complex procedures involved in pancreatic surgery. Length of operation > 8 h, MPD < 3 mm, and abdominal wall thickness > 10 mm, were found to be independent risk factors for superficial SSIs [7]. Other factors include age > 70, the presence of biliary stones, preoperative jaundice, malnourishment, malignancy, and surgery in the emergency setting [9]. All these factors may contribute to increased SSI but are not modifiable.

In a few cases of periampullary tumors undergoing pancreaticoduodenectomy (PD), we observed that by modifying the sequence of steps of the operation so that the bile duct is divided only after the completion of the pancreatojejunostomy (PJ) anastomosis, the incidences of SSI were less. However, no published studies have yet concluded that there is an advantage in dividing the bile duct in the last step compared to the standard procedure of dividing the bile duct early in the dissection process. Hence, we designed this prospective cohort study using a modified sequence pancreaticoduodenectomy to estimate the incidences of SSI and explore other advantages.

## 2. Materials and Methods

We designed this prospective cohort study to elucidate the incidence of SSIs in patients undergoing pancreaticoduodenectomy (PD) through the modified steps we propose for periampullary malignancy. The secondary objectives include an assessment of the postoperative return of bowel activity, the difference in length of hospital stay, added surgical advantages, and the incidence of postoperative morbidity and mortality. We coined the modified technique COMBILAST as an acronym for the sequence of Whipple’s procedure’s “common bile duct last” approach. 

In the study, we included patients undergoing Whipple’s pancreaticoduodenectomy for a periampullary malignancy in a single surgical unit at a tertiary teaching hospital. We excluded patients who declined to participate in the study, had a proven postoperative leak from the pancreatojejunostomy, hepaticojejunostomy, or gastrojejunostomy site, or had a Grade B or C postoperative pancreatic fistula. Before recruiting patients, we obtained approval from the Institutional Ethical Committee. This study was conducted between June 2018 and December 2021.

All patients underwent the essential preoperative workup and received preoperative optimization. Patients with the resectable disease posted for Whipple’s procedure underwent the same using the COMBILAST sequence. Patients with cholangitis, coagulopathy, and intractable pruritus underwent preoperative biliary drainage (PBD) through endoscopic stenting (EBD). We sent bile for culture in these patients, and antibiotics were started based on the sensitivity report. These patients were then discharged with antibiotics and other necessary medications once the infective complications subsided and the metabolic derangements had started to reverse; they were advised to follow up after four weeks after undergoing a pancreaticoduodenectomy [11]. Patients without any pre-existing positive bile culture in their report received an empirical preoperative injectable antibiotic dose of cefuroxime 1.5 g and metronidazole 500 mg within an hour before the skin incision. Based on their past bile culture reports, those with stents were started on antibiotics the previous night, and another dose was repeated within an hour of the skin incision. The antibiotics were repeated intraoperatively based on the drug’s half-life. Post-operatively, the antibiotic coverage was continued for 72 h, by which time the intra-operative bile culture results were obtained. The antibiotics were stopped in patients with a negative culture and if they did not have any other obvious infective complications. In case of a positive bile culture, the antibiotic coverage was continued for a period of 7 days based on the culture report. The antibiotic was then tapered or altered based on the patient’s overall condition.

An Ioban^®^ (3M^TM^) antimicrobial incise drape was routinely applied after recommended skin preparation with an antiseptic solution (chlorhexidine) to prevent surgical wound contamination with skin microbes. In the classical Whipple operation, after assessing the tumor resectability status, the distal common hepatic duct (CHD) is divided just proximally to the cystic duct entry site in the early part of the operation. After that, the entire procedure is completed with the division of the stomach, pancreas, and jejunum. The reconstruction procedure is followed in the sequence of pancreatojejunostomy (PJ), hepaticojejunostomy (HJ), and gastrojejunostomy (GJ). The transected bile duct is either left open to drain freely into the peritoneal cavity or intermittently clamped with a Satinsky clamp for a few hours until the HJ anastomotic procedure is over.

**Figure 1:**
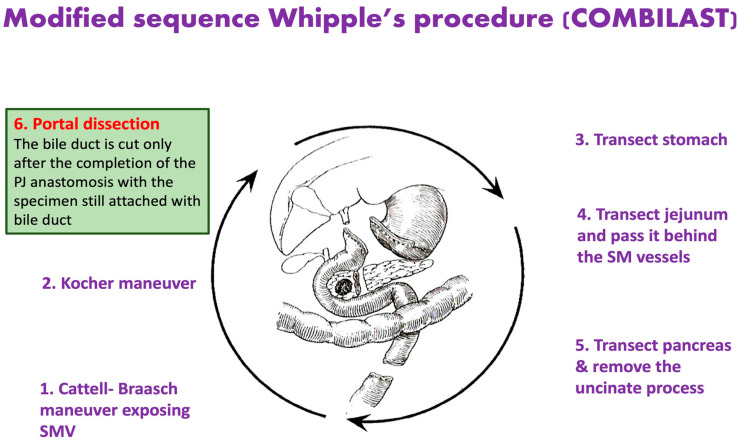
Modified Sequence Whipple’s Procedure, where the common bile duct is divided only after completing the pancreatojejunostomy anastomosis.

**Figure 2:**
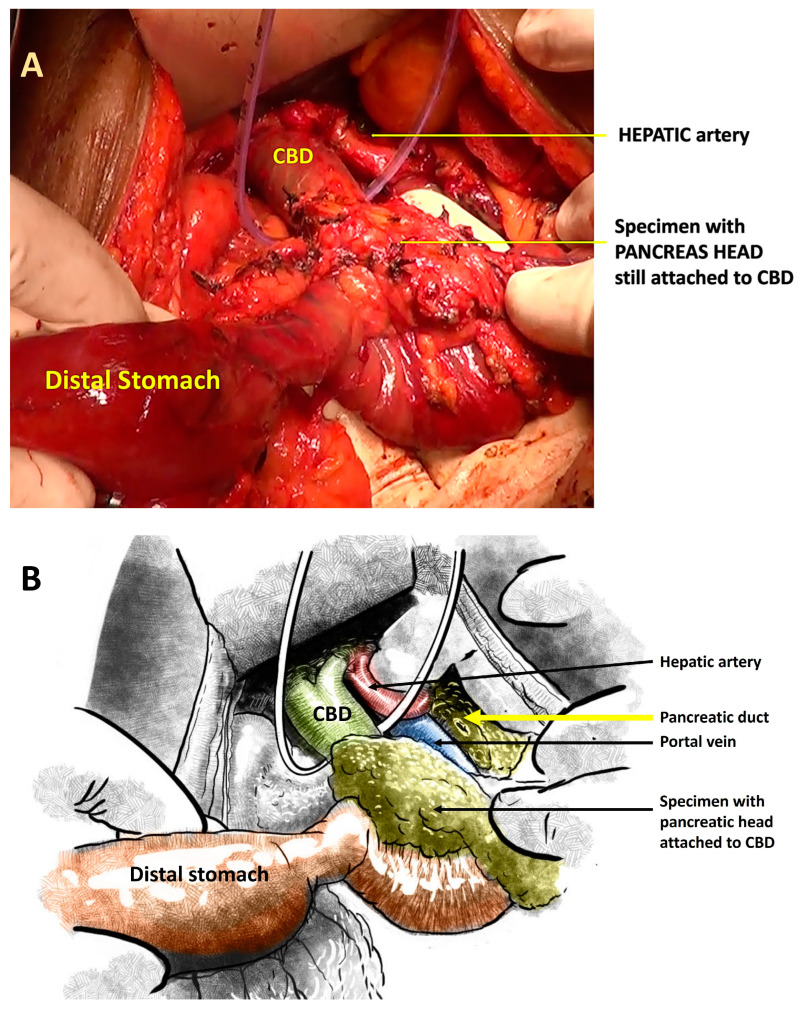
(**A**): Intraoperative photograph showing the specimen where the pancreatic head is still attached to the CBD. (**B**): Schematic illustration of the same.

**Figure 3:**
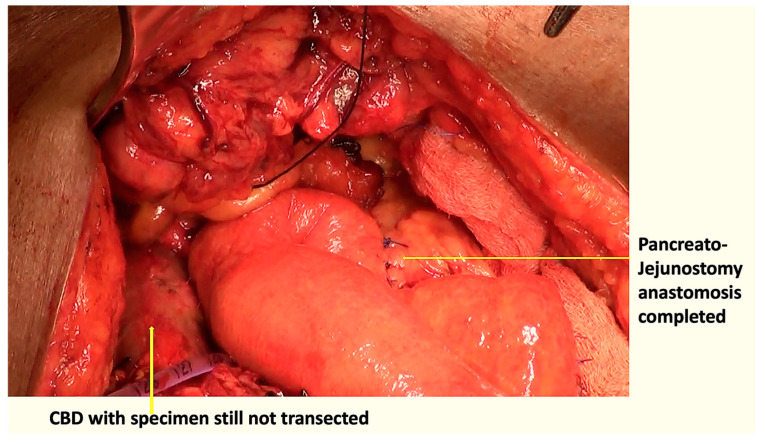
Pancreatojejunostomy anastomosis completed with the specimen still attached to the CBD.

**Figure 4:**
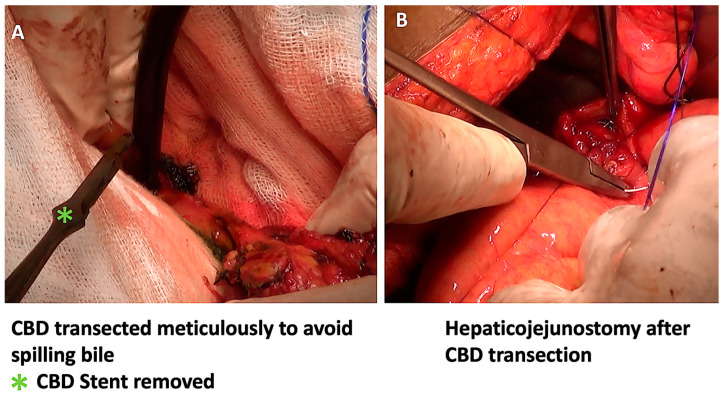
After the PJ anastomosis, the CBD was divided with minimal bile spillage (**A**), and the hepaticojejunostomy anastomosis was completed (**B**).

In the modified sequence COMBILAST technique we propose, the bile duct was divided only after completing the PJ reconstruction instead of an early division (Figure 1, Figure 2, Figure 3 and Figure 4). Hence there was minimal biliary spillage into the peritoneal cavity and contamination of the abdominal wound with bile from the obstructed biliary system, which we assumed was one of the preventable factors responsible for SSI. Intraoperative bile cultures were obtained in all patients after the transection of the hepatic duct. 

The specimen, while still attached to the bile duct, was flipped over the wound margins onto the anterior abdominal wall and lower chest so as not to compromise the operative space. One needs to be careful from this point on to avoid excess traction on the specimen and an inadvertent avulsion of the bile duct.

We monitored all the patients in the postoperative period for any procedure-related complications, especially SSIs, pancreatic fistulas, and anastomotic leaks. The CDC guidelines were followed to diagnose and classify SSIs [12]. In the event of an SSI, we sent a wound swab for culture from all patients. The bile culture isolates from the same patient were compared to the wound isolates to look for common organisms. All other complications were diagnosed and managed per guidelines [13,14,15,16].

Continuous variables are expressed as the mean and standard deviation (SD) for normal distributions or the median and interquartile range (IQR) for skewed distributions. Categorical variables were expressed as percentages. Statistical analysis was performed in SPSS 25.

## 3. Results

**Figure 5:**
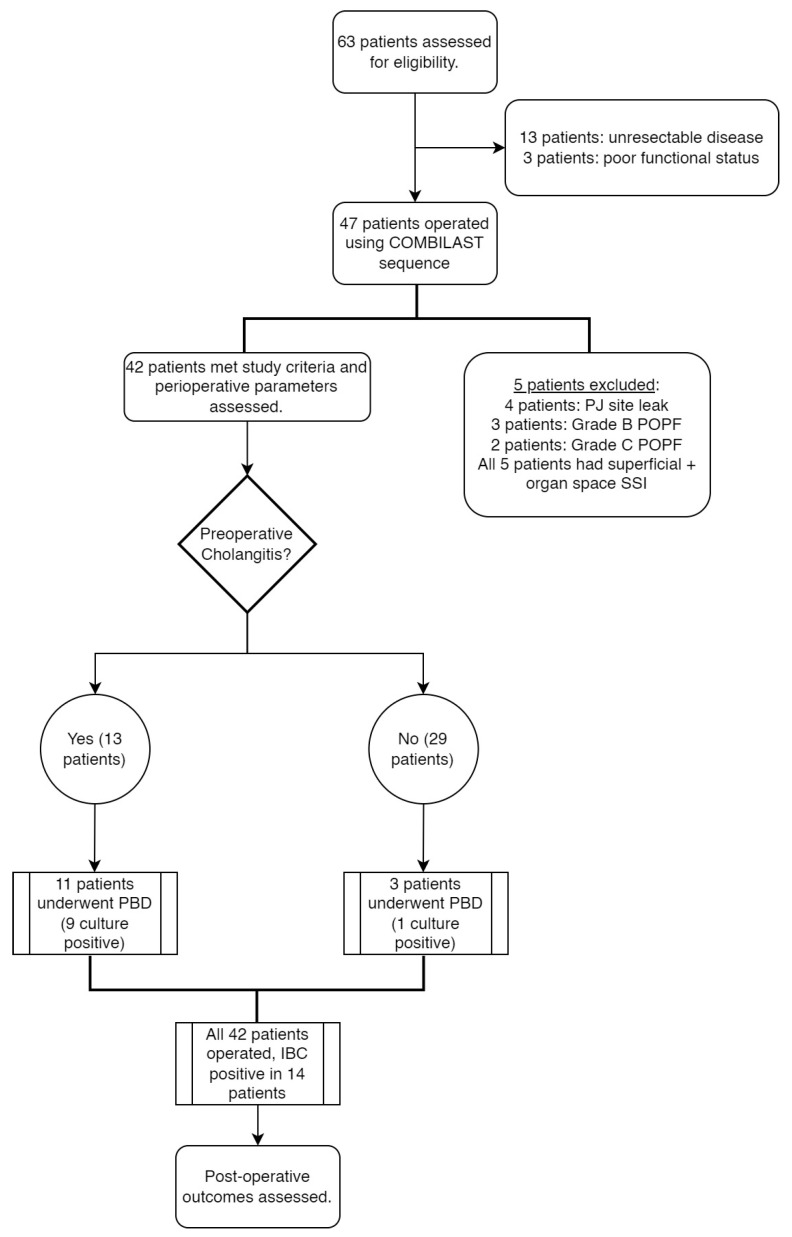
Flow diagram of the study.

During the study period, we assessed 63 cases for operability. Of them, 13 patients had an unresectable disease based on preoperative imaging or a staging laparotomy, and three were deferred surgery due to poor functional status. A total of 47 patients underwent the Whipple procedure using the COMBILAST sequence. We excluded five patients from the study because of postoperative anastomotic leaks and pancreatic fistulas. A total of 42 patients were considered for the final analysis. The flow diagram of our study is shown in Figure 5.

### 3.1. Preoperative Parameters

The study population had a mean age of 58.34 ± 10.24 years and a sex ratio (F:M) of 26:16. The mean BMI was 22.97 ± 3.42 kg/m^2^. Most patients had a functional status of ECOG 1 (38.1%) or 2 (35.7%). The median symptom duration was 74 days. Of the study population, 35.7% were hypertensive, while 33.3% were diabetic, and 28.6% of the patients had a history of smoking. Most of the patients (40.5%) fell into the ASA 1 category. The preoperative parameters are summarized in Table 1.

**Figure 6:**
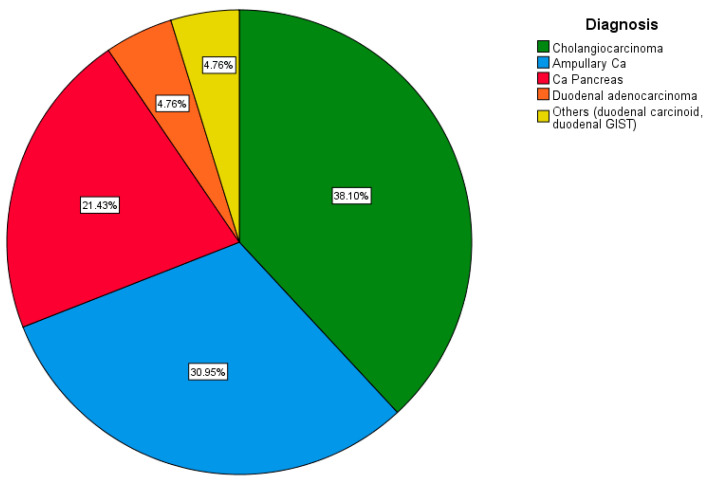
Pie chart depicting the distribution of cases based on diagnosis.

A total of 16 (38.1%) patients were diagnosed with distal cholangiocarcinoma, and another 13 (30.9%) were diagnosed with ampullary carcinoma confirmed on an upper GI endoscopy. The remaining nine (21.4%) were diagnosed with a lesion in the head of the pancreas, two (4.8%) with duodenal adenocarcinoma, and one each (2.4%) with a gastrointestinal stromal tumor (GIST) and carcinoid of the duodenum (Figure 6). 

The mean bilirubin level of the study population was 7.88 ± 5.78 mg/dL. Five (11.9%) patients had preoperatively raised liver enzymes. A total of 13 (30.9%) patients had preoperative cholangitis, and 11 of them required preoperative biliary drainage with stenting. Another three patients underwent preoperative biliary drainage due to intractable pruritus and coagulopathy. Out of all the 14 patients undergoing preoperative biliary stenting, 10 (71.4%) patients had a positive bile culture. Eight out of ten patients, or 80%, had a positive preoperative bile culture and an antibiogram with sensitivity to piperacillin and tazobactam; the rest had sensitivity to carbapenems.

### 3.2. Operative Details

The mean operative duration was 391.28 ± 67.86 patients, and the mean blood loss was 705 ± 172 mL (Table 2). 

**Figure 7:**
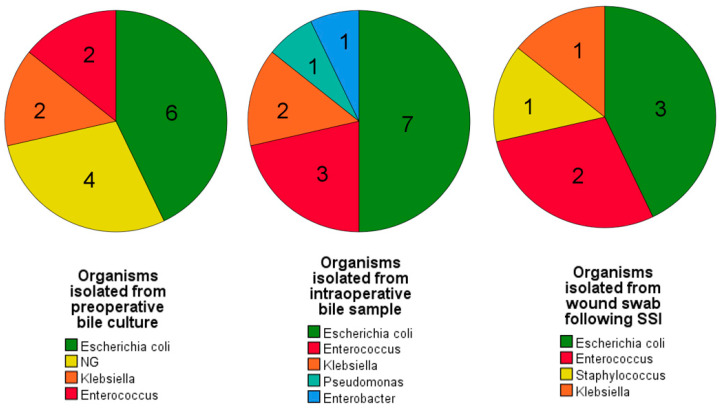
Pie charts depicting the organisms that showed predominant growth following culture from preoperative bile samples, intraoperative bile samples, and postoperative wound swabs. A similar growth pattern is evident among all three cultures. [NG = No growth].

Intraoperative bile culture (IBC) was positive in 14 (33.3%) patients. The rate of IBC positivity was higher in those who underwent preoperative biliary drainage. Even though most of the cultures were polymicrobial, *Escherichia coli* was the most common organism isolated (Figure 7).

### 3.3. Postoperative Outcomes

In the postoperative period, seven (16.7%) patients developed a superficial surgical site infection, and two (4.8%) patients had an additional deep SSI. Of the seven patients, four patients (57.14%) had undergone preoperative stenting. IBC had a higher positivity rate in patients who had undergone PBD (64.3%) vs. those who had not (17.9%). Six (85.7%) of these patients also had a positive IBC, and the IBC matched the SSI isolate in five (71.4%) patients (Table 3).

*Escherichia coli* was the most common organism isolated from wound swabs (Figure 7).

A total of 14 (33.3%) patients had a Clavien–Dindo grade of III or higher postoperative surgical complications. Of them, twelve (28.6%) had a postoperative collection, nine (21.4%) patients suffered from septicemia, and four (9.5%) patients required re-exploration. Three (7.1%) patients died of septicaemia from postoperative complications. The average length of hospital stay was 13.00 ± 5.92 days (Table 4). 

Out of all the 47 patients operated on under the COMBILAST sequence, four (8.5%) patients had PJ site leaks. A Grade B pancreatic fistula was observed in three patients (6.38%) and a Grade C POPF in two patients (4.2%). 

**Figure 8:**
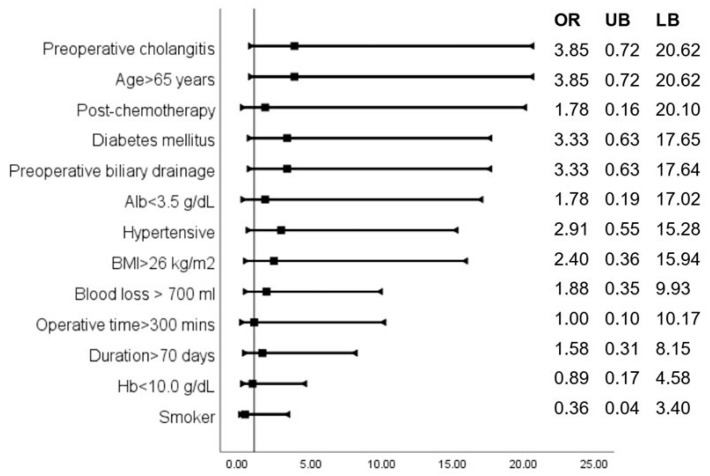
Odds ratios indicate the association of various parameters with the occurrence of SSIs.

Based on risk analysis, a positive intraoperative bile culture had the highest association with the occurrence of SSI (OR: 20.25, 95% CI: 2.12, 193.91), followed by the male gender (OR: 5.46, 95% CI: 0.91, 32.62). The odds ratios of other factors likely to be associated with SSIs are shown in Figure 8.

### 3.4. Additional Findings

**Figure 9:**
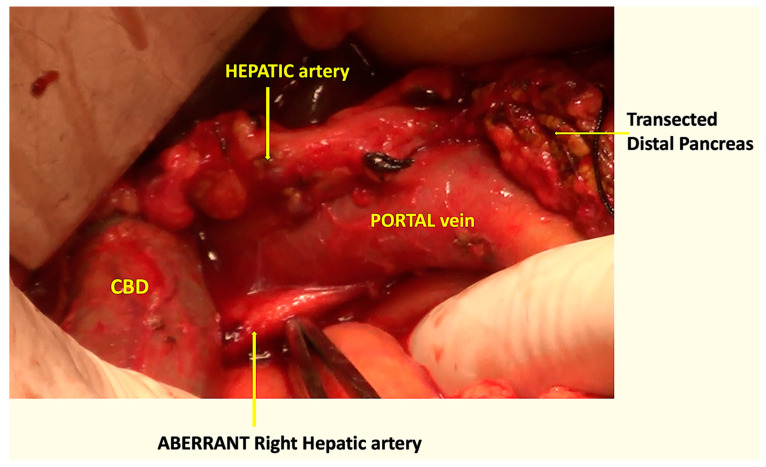
Intraoperative photograph showing the aberrant right hepatic artery, passing behind the portal vein and the CBD.

An aberrant, replaced, or accessory right hepatic artery could be identified in eight (17.0%) cases (Figure 9). By using the COMBILAST approach after the division of the pancreatic head, while dissecting the bile duct from the portal vein from below upwards, we could quickly identify the aberrant vessels and prevent them from accidental injury. The preoperative radiological investigations identified the presence of unusual vessels in only half of the cases.

## 4. Discussion

Based on our modified sequence of Whipple’s procedure, we experienced a significantly lower SSI rate (16.67%) compared to the reported incidences of >20% [1,4,17,18,19]. In our center, as per the monthly audit report and our databases, the SSI rate after PD is about 33% if performed without the COMBILAST sequence and in the absence of a PJ anastomotic leak. Our technique probably reduces biliary contamination and effectively prevents the conversion of a Class II surgical field to a Class III. As evident from our longitudinal study and reflected in pre-existing literature, most organisms isolated from surgical site infections are the same as the intraoperative bile culture isolates. Thus, biliary contamination is an essential factor contributing to SSIs. As per Coppola et al., a positive biliary culture was found in 66% of patients undergoing PD, which led to an increase in postoperative complication rates, including SSIs [20]. Despite the COMBILAST approach, we still had SSIs, even with taking the utmost care to prevent contamination from the spilt bile; nonetheless, inadvertent contamination of the bile with the wound must be the reason for the reduced incidence rate. Our technique, combined with additional new recommendations such as a prophylactic NPWT dressing and wound protectors, refs. [5,6,19,21] may further reduce the SSI rates. Duodenoscopic surveillance to prevent patient colonization through contaminated instruments during ERCP, as recommended by Cicozzi [22], is an additional strategy to reduce the rate of bacterobilia.

Various factors, such as a positive IBC, male gender, preoperative cholangitis, age > 65 years, diabetes, and preoperative biliary drainage, are identified and associated with an increased risk of SSIs [19]. The presence of one or more of these factors could help in the identification of high-risk populations.

As per few studies, PBD has no effect on the incidence of significant post-operative complications after pancreaticoduodenectomy [23,24]. However, contradicting results have been reported by other authors [25]. In our study, we found an odds ratio of 3.33 for the development of SSIs in patients who had undergone preoperative biliary drainage. All 14 patients underwent preoperative biliary drainage through ERCP. 

The presence of a post-operative PJ leak or fistula is known to be a significant risk factor for incisional and organ space SSI [18]. For this reason, we excluded the five patients with a postoperative PJ leak or POPF, which could cause a bias in ascribing the exact cause of SSIs sought in our study.

The other risk factors for the development of SSIs, as suggested by Katherine et al., are neoadjuvant chemotherapy and radiation, operative time greater than 7 h, intraoperative red blood cell transfusion, and concomitant vascular resection [25].

By modifying the steps, we found the advantage of quickly identifying an accessory or aberrant right hepatic artery near its origin while dissecting the bile duct from below upward. In our study, we identified such variations of the arterial anatomy in eight (17%) cases, and there was no incidence of injury to the vessels. In the classical approach of the Whipple procedure, the bile duct is divided early by creating a plane between and the portal vein at the hepatic duct level. If the aberrant vessels are missed during the radiologist’s preoperative assessment of the CT angiography, then, in those cases, there are increased chances of inadvertent transection of the vessels while dividing the bile duct early. Unfortunately, we missed the finding in four cases. However, using the COMBILAST sequence, an injury was avoided. Inadvertent injury to the replaced right hepatic artery may result in bleeding complications, liver or bile duct ischemia, and subsequent breakdown of the bilio-enteric anastomosis [26].

Another advantage of the COMBILAST approach is the avoidance of a bulldog clamp to the cut end of the hepatic duct for a prolonged period time, as in a classical approach. It can avert the apprehension of possible ischemic injury to the cut end of the duct with the chance of a hepaticojejunostomy leak.

We had a mean operating time of 391.28 ± 67.86 min and a median blood loss of 705 mL, comparable to those in existing literature [5,18,19,27,28]. Fourteen (33.3%) patients had a Clavien–Dindo grade of III or more, again in conjunction with existing evidence [4]. The rates of Grade B or C postoperative pancreatic fistula (10.6%), re-exploration (9.5%), and length of hospital stay (13.00 ± 5.92 days) also were within the reported ranges. Thus, modification of the sequence did not adversely affect the operative duration or blood loss, nor did it cause an increase in postoperative complications. 

There is always a theoretical concern about the release of toxins released from the ischemic specimen into the blood stream. However, we did not find any significant difference in the mean post-operative pH or lactate levels between patients operated on with and without the COMBILAST sequence. The probable explanation could be the complete devascularization of the specimen, leaving only the bile duct intact.

Our institute is not an exclusive center for pancreatic surgery. Hence, the number of cases operated on is relatively low, which is one of our study’s limitations. The average rate of hospital-acquired infections is reported to be higher in the Indian population compared to CDC-reported rates, and hence infectious complications are expected to be higher [29]. Therefore, we encourage further studies and randomized controlled trials with a good sample size to evaluate the efficacy and safety of the COMBILAST technique.

## 5. Conclusions

Surgical site infections (SSIs) following pancreaticoduodenectomy significantly cause morbidity and mortality. The modified sequence of the Whipple procedure, by the COMBILAST (common bile duct division last) technique, seems to be promising in reducing the SSIs and the hospital stay of the patient, thereby reducing the economic burden on the patient and the health system. As the approach is only a modification of the operative sequence, compromises in oncological safety are not expected. It has an added surgical advantage in reducing the chance of injury to the aberrant or accessory right hepatic artery. 

## Figures and Tables

**Table 1:** Preoperative characteristics of the study population.

Parameter	Overall (*n* = 42)
Age (years) (mean, SD)	58.34, 10.24
Sex (F: M)	26:16
BMI (kg/m^2^) (mean, SD)	22.97, 3.42
ECOG: *n* (%)	
0	11 (26.2%)
1	16 (38.1%)
2	15 (35.7%)
Symptom duration (median, IQR)	74, 132
Comorbidities: *n* (%)	
Diabetes	14 (33.3%)
Hypertension	15 (35.7%)
Alcohol intake	5 (11.9%)
Post chemotherapy	4 (9.5%)
Smoker	12 (28.6%)
ASA: *n* (%)	
1	17 (40.5%)
2	15 (35.7%)
3	10 (23.8%)
Preoperative cholangitis: *n* (%)	13 (30.9%)
Variant of periampullary carcinoma: *n* (%)	
Cholangiocarcinoma	16 (38.1%)
Carcinoma of the head of the pancreas	9 (21.4%)
Ampullary carcinoma	13 (30.9%)
Duodenal adenocarcinoma	2 (4.7%)
Duodenal GIST	1 (2.4%)
Duodenal carcinoid	1 (2.4%)
Hb (g/dL) (mean, SD)	10.26, 1.2
TLC (1000/µL) (mean, SD)	11.46, 3.75
Albumin (g/dL) (mean, SD)	3.06, 0.81
TB (mg/dL) (mean, SD)	7.88, 5.78
Transaminitis: *n* (%)	5 (11.9%)
Preoperative biliary drainage: *n* (%)	14 (33.3%)

**Table 2:** Intra-operative parameters.

Parameter	Overall (*n* = 42)
Operative duration (min) (mean, SD)	391.28, 67.86
Blood loss (mL) (median, IQR)	705.13, 172.97
Bile duct diameter (cm) (mean, SD)	15.28, 5.81

**Table 3:** Details of the cases with SSIs, along with the antibiotic sensitivity profile.

Parameter	(*n* = 42)	
**SSI**: *n* (%)		
Overall	7 (16.7%)	
Superficial	7 (16.7%)	
Deep	2 (4.8%)	
**SSI isolates**: *n* (%)		**Antibiotic Sensitivity Profile:**
*Escherichia coli*	3 (42.85%)	Piperacillin-tazobactam (2), Gentamicin (2), Amikacin (3), Imipenem (3)
*Enterococcus*	2 (28.57%)	Ampicillin (1), Amoxyclav (2), Linezolid (2), Vancomycin (2)
*Klebsiella*	1 (14.28%)	Piperacillin-tazobactam, Amikacin, Imipenem
*Staphylococcus*	1 (14.28%)	Linezolid, Vancomycin
Wound isolate **matched** intraoperative biliary isolate in: *n* (%)	5 (71.4%)	

**Table 4:** Other postoperative outcomes.

Parameter	Overall (*n* = 42)
Postoperative pancreatic hemorrhage: *n* (%)	3 (7.1%)
Chyle leak: *n* (%)	3 (7.1%)
Collection: *n* (%)	12 (28.6%)
Pneumonia: *n* (%)	12 (28.6%)
UTI: *n* (%)	4 (9.5%)
Septicemia: *n* (%)	9 (21.4%)
Reexploration: *n* (%)	4 (9.5%)
Mortality: *n* (%)	3 (7.1%)
Clavien–Dindo Grade: *n* (%)	
2	28 (66.7%)
3	8 (19.0%)
4	3 (7.1%)
5	3 (7.1%)
Length of hospital stay (days): mean, SD	13.00, 5.92

## Data Availability

The data of the recruited patients are shared in the manuscript, and details are available with us. The corresponding author can be contacted for any further data.
